# Nutritional Support in Coronavirus 2019 Disease

**DOI:** 10.3390/medicina56060289

**Published:** 2020-06-12

**Authors:** Ewa Stachowska, Marcin Folwarski, Dominika Jamioł-Milc, Dominika Maciejewska, Karolina Skonieczna-Żydecka

**Affiliations:** 1 Department of Human Nutrition and Metabolomics, Pomeranian Medical University in Szczecin, 71-460 Szczecin, Poland; dominika.jamiol@pum.edu.pl (D.J.-M.); dmaciejewska.pum@gmail.com (D.M.); karzyd@pum.edu.pl (K.S.-Ż.); 2 Department of Clinical Nutrition and Dietetics, Medical University of Gdansk, 80-210 Gdańsk, Poland; marcinfol@gmail.com; 3 Home Enteral and Parenteral Nutrition Unit, Nicolaus Copernicus Hospital, 80-803 Gdańsk, Poland

**Keywords:** COVID-19, SARS-CoV-2, coronavirus, nutrition support, nutrition

## Abstract

The epidemic that broke out in Chinese Wuhan at the beginning of 2020 presented how important the rapid diagnosis of malnutrition (elevating during intensive care unit stay) and the immediate implementation of caloric and protein-balanced nutrition care are. According to specialists from the Chinese Medical Association for Parenteral and Enteral Nutrition (CSPEN), these activities are crucial for both the therapy success and reduction of mortality rates. The Chinese have published their recommendations including principles for the diagnosis of nutritional status along with the optimal method for nutrition supply including guidelines when to introduce education approach, oral nutritional supplement, tube feeding, and parenteral nutrition. They also calculated energy demand and gave their opinion on proper monitoring and supplementation of immuno-nutrients, fluids and macronutrients intake. The present review summarizes Chinese observations and compares these with the latest European Society for Clinical Nutrition and Metabolism guidelines. Nutritional approach should be an inseparable element of therapy in patients with COVID-19.

## 1. Introduction

Coronavirus (CoV) infections have been a major public health concern for almost two decades. The very first corona-viral epidemic outbreak took place in 2002 in China and was linked to severe acute respiratory syndrome—the entity that gave the name for this viral agent, SARS-coronavirus (SARS CoV). A second coronavirus outbreak with similar symptoms spread throughout Middle East in 2012. Consequently the virus name was MERS-CoV. During these two epidemics, as many as 10,500 cases were confirmed with the mortality rate of about 15% [1].

On 11 February 2020, the World Health Organization (WHO) announced the epidemic outbreak of the novel coronavirus family members, SARS CoV-2. A month later, the status was pandemic. The virus was reported to be very contagious and has spread globally in a short period of time, as the infections seem to have originated in China, in December 2019 [1]. As for the end of March 2020, WHO reported about 450,000 confirmed cases of infection and 19,000 deaths in almost 200 countries around the world [2].

The clinical features of SARS-CoV-2 infection, namely a coronavirus disease 2019 (COVID-19), range from asymptomatic to severe conditions including respiratory and multiorgan failure. Major symptoms are fever, dry cough, fatigue, myalgia, and dyspnea. Headache, hemoptysis and diarrhea occur less commonly [3,4,5,6]. Medical protocol consists mainly of symptomatic treatment and the prevention of secondary infection [7]. Effective antiviral treatment for SARS-CoV-2 has not been identified yet. The antiviral drugs, including ganciclovir, oseltamivir, ritonavir, ribavirin, and lopinavir have been used to prevent the likelihood of respiratory complications in several studies, but the efficacy awaits to be verified within coming months [7].

The data on nutritional support in COVID-19 patients is still elusive. This refers to the nutritional status of the patients, including as well mild to severe cases, and most importantly the malnutrition that enhances the probability of poor outcome [8,9]. This work summarizes what has already been done in the field and drawn some general recommendations for the dietary management in coronaviruses treatment. Viral etiology of community-acquired pneumonia (CAP) may by underdiagnosed. As it was shown, about 8% of cases are due to influenza virus infection, whilst the lowest proportion might be caused by rhinovirus, coronavirus and respiratory syncytial virus. The odds of death during a hospital stay were shown to be significantly higher in case of both bacterial and viral infections [10]. Consequently, due to lack of specific COVID-19 data, the recommendations in our review are based on viral and bacterial pneumonia studies, as well as recommendations made for critically ill patients.

### Method

Literature review was conducted by all authors. Studies published until 27/03/2020 were included. Search words were: (“coronavirus” OR “severe acute respiratory syndrome coronavirus 2” OR “severe acute respiratory syndrome coronavirus 2” OR “sars cov 2” OR”SARS-CoV-2” OR “COVID-19” OR “severe acute respiratory syndrome coronavirus 2”) AND (“Nutrition” OR “Diet” OR ”Home nutrition” OR “Enteral nutrition” OR “energy expenditure” OR “Parenteral nutrition” OR “probiotics” OR“pneumoniae” OR “sepsis” OR “intensive care” OR “critical illness” OR “Nutrition” OR “Diet” OR ”Home nutrition” OR “Enteral nutrition” OR “energy expenditure” OR “Parenteral nutrition”).

The articles included were, however, not selected on a systematic basis, thus the evidence reviewed might not be exhaustive. Summarized data were analyzed together with existing recommendations for nutritional interventions published by ESPEN and ASPEN and reviewed in perspective of COVID-19 pandemic. 

## 2. Nutritional Treatment

### 2.1. Nutrition in Mild COVID-19 Infection

**Figure 1:**
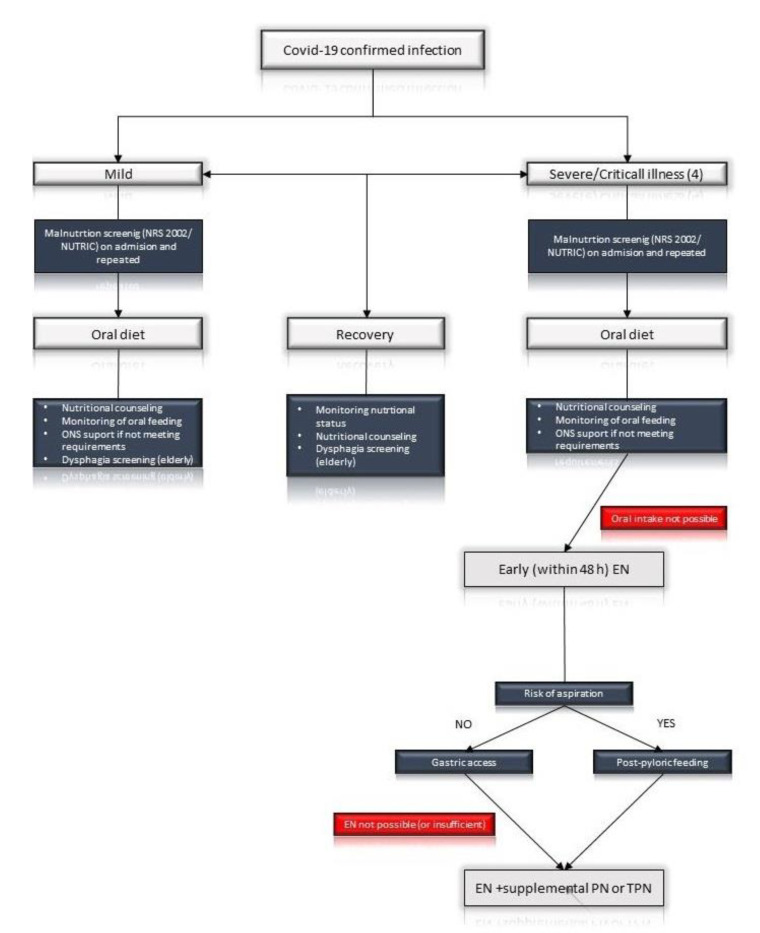
Simplified schema according to: “ESPEN guideline on clinical nutrition in the intensive care unit [16]”. TPN—Total Parenteral Nutrition, EN—Enteral Nutrition, PN—Parenteral Nutrition, ONS Oral—Nutritional Supplement.

Data concerning patients with CAP show that 39% of persons admitted to hospitals are malnourished and their nutritional status may serve as both short and long-term prognostic factord of mortality especially among the elderly [11]. To add, low albumin level was associated with higher 30-day and 2.5-year mortality rates [12]. Age and preexisting comorbidities like cardiovascular disease, diabetes, chronic respiratory disease, hypertension, and cancer were found to be linked to higher case-fatality rate in COVID-19 pneumonia [8,9]. Moreover, the clinical status of patients with mild symptoms at the time of diagnosis may deteriorate during the observation time. Consequently, we advise nutritional screening including mild cases of COVID-19 infections. Medical staff should monitor effective oral nutrition and are encouraged to prescribe oral nutritional supplements (ONS) for patients who are not able to cover the energy and protein requirements with fortified meals. Elderly patients should be repeatedly screened and advised to fill in a dietary recall diaries. This should also apply for nursing and long term facilities, as significant prevalence of malnutrition in home-care services have been described [13]. Moreover, screening for dysphagia in prehospital setting and after hospital admission is advised. Oropharyngeal dysphagia in patients with pneumonia has been correlated with higher mortality and worse long-term outcome [14]. Easy and validated questionnaires could be useful for swallowing disorders screening. For instance, “Eat-10” survey, which does not require direct contact with a patient and can be done using telemedical devices [15] (Figure 1).

### 2.2. COVID-19-Specific Recommendations for Home-Nutrition Units

Patients receiving home parenteral nutrition (HPN) and home enteral nutrition (HEN) are at risk of the severe course of the COVID-19 infection, predominantly due to coexisting diseases. To our knowledge, there are no publications concerning modification of nutritional treatment in HPN or HEN during the Covid-19 pandemic. However, sanitary and epidemiological issues during a pandemic should be addressed at the same time as nutrition-related procedures. Home care units should limit personal visits and contacts with patients to life-threatening circumstances. Patients should be under continuous monitoring using telemedical solutions. Hospital treatment should be considered if any signs of infection are noted. Family and care holders are to follow strict epidemiological guidelines, which is a potential source of infection for the patients.

### 2.3. Probiotics

No clinical trials showed the benefits of probiotic usage in COVID-19 infection. However, several studies underlined a possible relation between lung microbiome and pulmonary diseases. The hypothesis of the gut–lung axis refers to a link between diet and bacterial metabolites like short chain fatty acids (SCFA) and lung microecological niche [17]. Experimental studies in animals have demonstrated the protective role of trans nasal administration of *Lactobacillus* species against influenza infection [17,18,19]. There are no precise guidelines for microbial agents to use in the critically ill, however, a number of studies have shown potential benefits of such administration in the intensive care unit (ICU) setting [20,21]. COVID-19 patients on antibiotic therapy with gastrointestinal symptoms may be the target group for probiotic supportive therapy. Above-mentioned data, however, provide insufficient data to recommend probiotic use in Covid-19 infection. Experimental studies are to show future directions for human trials.

### 2.4. Critically Ill Patients 

Nutritional treatment for critically ill patients diagnosed with COVID-19 (especially in case of respiratory and multiorgan failure) [22] is a key element of comprehensive treatment aimed to reduce the mortality. The observations made by Chinese nutritionists might help to improve the efficacy of nutrition-based approaches in COVID-19 patients [23].

The development of malnutrition among critically ill COVID-19 patients is caused by imbalances in energy intake and expenditure secondary to:

(1) increased energy consumption due to fever, mechanical ventilation, exacerbated activity of breathing muscles, and hyper catabolism.
(2) insufficient intake of nutrients due to decline in appetite, dyspnea, mechanical ventilation, and disturbance of consciousness. In case of direct attack of coronavirus on the gastrointestinal tract, nausea, diarrhea, or vomiting caused either by enteral nutrition intolerance or antiviral drug treatment might occur [23].
(3) Metabolic disorders involving (a) Impaired glucose metabolism (increased blood sugar and insulin resistance, reduced glucose oxidation, increased glycolysis and gluconeogenesis); (b) impaired protein metabolism (increased protein breakdown, and enhanced synthesis of acute phase proteins, decreased muscle protein synthesis and negative nitrogen balance in the body); (c) increased fat mobilization and decomposition.

### 2.5. Nutrition Screening for Critical Patients 

For critically ill patients, dynamic nutritional risk screening is recommended. The assessment and the setting of nutritional support objectives should be conducted during admission to the ICU and during the implementation of enteral nutrition, during the first 24–48 h [22]. A meta-analysis conducted in ICU patients demonstrated that early enteral nutrition within 24 h of ICU admission reduced mortality compared with delayed enteral intake (odds ratio, 0.45; 95% CI, 0.21–0.95, *p* = 0.038) [24].

The evaluation of malnutrition should be repeated regularly and frequently due to the dynamics of the disease and increased risk of dysphagia in the elderly after pneumonia, and after prolonged respiratory therapy (post-extubation dysphagia) [25,26,27]. Nutrition Risk Screening Form (NRS 2002) [28] is a recommended tool. A modified NUTRIC score is recommended for screening [23] whenever history of body weight and dietary habits in critically ill patients are available [29]. The basic recommendations following these surveys are as follows:

NRS: score ≥ 3 points, nutritional risk intervention is needed; score ≥ 5 high nutritional risks, intervention as early as possible.

NUTRIC: ≥ 5 points (not considering IL-6), suggesting that patients have higher nutritional risk and need nutritional support as early as possible.

It is of high importance to remember that the nutritional status of critically ill patients may change rapidly, thus, this strategy should be ongoing. It is recommended to screen patients with low nutritional risk again after 3 days [23,30].

### 2.6. Nutritional Requirements

#### 2.6.1. Energy

The ideal method for measuring the actual energy consumption in human body is an indirect calorimetry (IC). However, reduced medical staff and high patient/nurse ratio in COVID-19 pandemic conditions limit the usage of IC. Therefore, in practice, the calculation the resting energy expenditure (REE) can be applied:

REE (kcal) = V_CO_2__ × 8.19 (1)

where the V_CO_2__ value should be measure by the ventilator.

Whenever it is impossible to measure V_CO_2__ directly, energy requirement needs should be estimated according to body weight:

- for non-obese critically ill patients, the recommended amount of energy is 25–30 kcal per day/kg
- for overweight and obese critically ill patients, the recommended amount of energy is 21 kcal per day/kg. Also the Penn State method (in ventilator-dependent obese patients) or HBE with actual body weight and a stress factor (1.1) (if the patient is spontaneously breathing) may be applied [31]
- For obese and critically ill patients, if the BMI is 30–50 kg/m^2^, the recommended energy target is 11–14 kcal/kg per day, for BMI > 50 kg/m^2^-22–25 kcal/kg per day (Table 1).

Ideal body mass should be calculated with Brock formula [32], but only for persons between 160 cm and 190 cm tall. Otherwise, the Hamwi formula is more sufficient [33].

*Brock formula*:

a) Men: Ideal body weight (kg) = height (cm) − 105,
b) Woman: Ideal body weight (kg) = [height (cm) − 100] × 0.85

*Hamwi formula*:

a) Men: 48.0 kg + 2.7 kg per 2.54 cm over 152.4 cm
b) Woman: 45.5 kg + 2.2 kg per 2.54 over 152.4 cm

*Penn State* [34].

Harris–Benedict: kcal/day = 0.85 (HBE) + 175 (*T*_max_) + 33 (Ve) − 6344.

Tmax, maximum temperature in a 24 h period; Ve, minute ventilation; HBE Harris–Benedict equation.

In patients in whom, due to fever/discomfort, reduced calorie intake was demonstrated, it is recommended to reach the target energy supply as soon as possible.

Typically, in critically ill patients, 20–25 kcal/kg/d is advised. According to European Society for Clinical Nutrition and Metabolism (ESPEN) guidelines and Chinese professionals, the initiation of nutritional support should be as soon as possible: 24 h/48 h after hospital admission starting with low dose energy for the first days [16]. Chinese doctors recommended the intake of 10–15 kcal/kg/day in the first few days [35]. Hypocaloric nutrition not exceeding 70% of estimated needs is advised, and 80–100% after 7 days.

Nutritional treatment might be implemented on the principles of five-step regimen for malnutrition [23], as demonstrated in Table 2.

Contraindications for EN include: uncontrolled shock, uncontrolled hypoxemia and acidosis, upper gastrointestinal bleeding, intestinal ischemia, intestinal obstruction, abdominal compartment syndrome, and high-output fistula without distal feeding pathways.

#### 2.6.2. Protein

Observational studies have shown that increasing the protein content in the diet of patients in critical condition can reduce their mortality, but it is still difficult to determine what is the optimal demand of a patient for protein [36]. For this reason, it is necessary to carefully observe side effects, evaluate the effects of treatment, and dynamically adjust the treatment plan toward COVID-19 clinical course. Protein supply should be about 1.5–2.0 g/(kg/day). When the protein supply does not cover the demand, a standard protein preparation is recommended [23]. 

#### 2.6.3. Fish Fatty Acids

To reduce oxidative stress and the incidence of acute respiratory distress syndrome (ARDS) and sepsis, a mixture of fish fats—docosahexaenoic acid (DHA) + eicosapentaenoic acid (EPA) might be used. For enteral feeding, 500 mg of EPA + DHA might be added daily for parenteral feeding from 0.1 to 0.2 g/(kg/d) [23].

Fluid therapy: It has been highlighted that most patients admitted to the ward are dehydrated due to high fever and loss of appetite at the beginning of the disease [22].

### 2.7. Gastric vs. Post-Pyloric Access

Gastric access is recommended by ESPEN [16] as the standard approach for EN (Table 3). Patients with high risk of aspiration require post-pyloric feeding. American Society for Parenteral and Enteral Nutrition (ASPEN) criteria for increased risk for aspiration are: inability to protect the airway, mechanical ventilation, age > 70 years, reduced level of consciousness, poor oral care, inadequate nurse: patient ratio, supine positioning, neurologic deficits, gastroesophageal reflux, transport out of the ICU, and use of bolus intermittent EN. Most of those characteristics are typical for COVID-19 patients with severe disease course [37]. Non-endoscopic methods of naso-jejunal or naso-duodenal tube placement are to be considered due to increased risk of COVID-19 transmission.

### 2.8. Vitamin D and Zinc

Vitamin D and zinc are nutrients that support optimal immune function [38]. Vitamin D receptors are present in many immune cells and modulate response to viral lung diseases reducing also the risk of respiratory infections [38]. Vitamin D deficiency is common in critically ill patients probably as a result of a reduction of Vitamin D Binding Protein (VDBP), and this has been associated with the increased ICU length of stay, mechanical ventilation and mortality [39,40,41]. 

Zinc is involved in the regulation of inflammatory responses through its influence on leukocytes and lymphocytes function, including their proliferation, differentiation and maturation and also has direct antiviral effect [42]. Although the data on direct antiviral effect of zinc on COVID-19 are limited, it has a significant impact on number of viruses involved in respiratory system pathology i.e. altering their replication [42]. 

The inflammatory process may influence intestinal mucosal integrity, causing malabsorption of essential nutrients and their blood concentration to decrease [43]. Many studies have indicated that zinc deficiency concerns 76–95% of patients at the time of ICU admission [43,44,45,46,47,48]. Consequently, we advise to estimate vitamin D and zink concentration in COVID-19 patients within the ICU and if necessary begin the supplementation. ESPEN recommendations concerning vitamin D and micronutrients administration in critically ill patients are presented in Table 3.

## 3. Conclusions

Covid-19 pandemic is a challenging health care problem. No specific antiviral interventions are effective to eliminate the COVID-19 infection, therefore, the multidisciplinary supportive medical approach is required to improve the outcome. Data concerning nutritional interventions for COVID-19 patients are still needed, nevertheless, conclusions from studies in severe viral and bacterial pneumonia could be integrated. Poor nutritional status is a prognostic factor for mortality in severe pneumonia and critical illness, especially for elderly patients. ESPEN and ASPEN guidelines for nutritional support in critical illness are applicable for COVID-19 patients requiring ICU support. Chinese experiences have shown that the medical approach is be adjusted for a low patient:medical staff ratio and war-like conditions in hospitals. Patients with a mild course of the disease should also be in the target of nutritional support, especially in advanced age or polymorbidity. Moreover, nutritional and dysphagia screening is advised for recovered individuals, and thus, long term outcomes of the infection have still not been analyzed. 

## Figures and Tables

**Table 1:** The strategy to measure energy supply regarding BMI.

BMI Value [kg/m^2^]	kcal/per day/kg Body Mass	Calculation of Ideal Body Mass (Y/N)	Calculation of Actual Body Mass (Y/N)
20–25	25–30	yes	
25–30	21		Yes
30–50	11–14		yes
> 50	22–25	yes	

**Table 2:** Nutrition-based strategies in critically ill patients that might potentially counteract COVID-19 clinical course [16].

Type of Patients	Strategy	Energy Protein Supply	Goal	Methods
Oral nutrition–possible and effective	Diet, Nutritional education	25–30 kcal/kg/day; 1.2–2.0 g/kg/day.	70% of nutritional requirements within 3 to 7 days	Encouraging patients to eat small meals In case of dysphagia-reducing The risk of aspiration (blending, semi-liquid diet)
Oral nutrition possible but not covering all requirements	diet ONS	25–30 kcal/kg/day; 1.2–2.0 g/kg/day.	70% of nutritional requirements within 3 to 7 days	400–600 kcal of energy through ONS (customized if necessary)
CIP who cannot eat by mouth (recommended for patients receiving ECMO)	TEN	10–20 mL/h or 10–20 kcal/h EN: adapted to coexisting diseases ^	Prevention of GI complications in mechanically ventilated Promotion of intestinal immune function; Maintaining the integrity of the intestinal barrier and function	Nasogastric tube to start EN High risk of aspiration—post-pyloric feeding
CIP contraindicated to EN or EN who cannot reach the target amount of energy ^^	PEN PN	Glucose initially up to 5 mg/kg/min; Fat: duration of infusion ≥ 8 h	When EN provide less than 60% of the target energy requirement	All-in-one preparation is recommended.

ONS-oral nutritional supplements, PN—total parenteral nutrition, TEN—total enteral nutrition, ECMO—ExtraCorporeal Membrane Oxygenation, CIP—critically ill patients, PEN—partial enteral nutrition, GI-gastrointestinal, ^ (a) conventional formula for patients with normal gastrointestinal function with no elevated blood glucose and renal insufficiency, (b) Diabetes formula (c) Kidney disease formula, (d) High energy density formula (1.5–2 kcal/mL), (e) High dietary fiber formula with soluble dietary fiber (fructo-oligosaccharide, inulin), amount: 10–20 g fiber in divided portions within 24 h, (f) Short peptide formula, (g) Pulmonary disease type formula (high fat/low carbohydrate); ^^ Severe malnutrition or high nutritional risk (NRS 2002 ≥ 5 points or NUTRIC score ≥ 5 points). Patients with mild symptoms should take their meals orally or with the support of an ONS.

**Table 3:** Nutrition therapy of critical ill patients, ESPEN recommendations [16].

Target	Patients Staying in the ICU, Mainly for More than 48 h (Considered at Risk for Malnutrition);
**Overall recommendations**	Careful and progressive re-introduction of nutrition;
	Severely malnourished and starved patients are at risk of refeeding syndrome;
	EN should be ceased in patients with uncontrolled shock, uncontrolled hypoxemia and acidosis, uncontrolled upper GI bleeding, gastric aspirate > 500 mL/6 h, bowel ischemia, bowel obstruction, abdominal compartment syndrome, and high-output fistula without distal feeding access ^1)^;
	Key points should be aimed for: (1) oral nutrition as early as possible while considering the risks of complications (e.g., Aspiration); (2) early EN at a low rate and progressive increase within 48 h if oral nutrition is not possible while considering the risk of complications; this progressive increase should be ruled by local protocols; (3) determination of the optimal starting point and dose of (supplemental) PN based on the risk of complications from oral or EN, state of acute illness and presence of previous under/malnutrition; ^1)^
**Nutritional risk determination**	Anamnesis, report of unintentional weight loss or decrease in physical performance before ICU admission;
	Physical examination;
	General assessment of body composition (if possible) to detect the loss of lean body mass and sarcopenia;
	Assessment of muscle mass and strength (if possible); considering the abovementioned parameters (the critically ill patient is compared to a geriatric patient);
	Main tools: grading of malnutrition according to the ESPEN GLIM recommendations (see Table 5, page 54, ESPEN guideline) or previous 2015 ESPEN criteria of malnutrition; Clinical Frailty Score (mainly in elderly patients);
	Additional tools: handgrip dynamometer (muscle function); Bioelectrical impedance (only in a stable patient not suffering from fluid compartment shifts);
	Criteria of malnutrition according to the 2015 ESPEN definition: BMI < 18.5 kg/m^2^ or suffering from an unintentional weight loss > 10% irrespective of time, or > 5% over the last 3 months combined with either a BMI < 20 if < 70 years of age, or < 22 if > 70 years old;
	Tools for screening the risk of malnutrition during hospital stay: the nutritional screening tools (NRS 2002); malnutrition universal screening tool (MUST); or pragmatic approach: patients staying in the ICU > 2 days, undergoing mechanical ventilation, infected, underfed > 5 days, and/or presenting with a severe chronic disease;
**Risk of overfeeding**	To avoid overfeeding, early full EN shall not be used but shall be prescribed within 3 to 7 days;
	In the early phase of critical illness the provision of excessive amounts of nutrients by any route should be avoided;
**Refeeding syndrome**	Energy supply should be restricted for 48 h and then progressively increased; measurements of electrolytes (potassium, magnesium, phosphate) during initiation of feeding is necessary to detect development of refeeding syndrome; refeeding hypophosphatemia is a warning signal (<0.65 mmol/L or a drop of > 0.16 mmol/L); electrolytes monitoring two to three times a day and supplemented if needed;
**Route and initiation time of nutrition therapy**	Oral diet preferred (patients who are able to eat) over EN or PN; if the patient is able to cover 70% of his needs from day three to seven, without risks of vomiting or aspiration;
	EN if oral intake is not possible; early EN (within 48 h) should be preferred over delaying EN and early PN; enteral feeding should be delayed when GRV is > 500 mL/6 h and application of prokinetics should be considered;
	PN should be implemented within 3 to 7 days if contraindications to oral and EN exist; low-dose PN (if EN is not feasible) should be implemented in patients with high nutrition risk (e.g., NRS 2002 ≥ 5) or severely malnourished because of the risks of overfeeding and refeeding;
**Type of access**	Gastric access-use as the standard approach to initiate EN;
	Postpyloric feeding-in patients with gastric feeding intolerance despite the use of prokinetic agents; postpyloric (mainly jejunal feeding) can be performed in patients at high risk for aspiration: inability to protect the airway, mechanical ventilation, age > 70 years, reduced level of consciousness, poor oral care, inadequate nurse:patient ratio, supine positioning, neurologic deficits, gastroesophageal reflux, transport out of the ICU, use of bolus intermittent EN; the small bowel feeding is associated with a reduced risk of pneumonia compared to gastric feeding;
**Administration of EN (bolus or continuous feeding)**	Continuous rather than bolus EN is recommended; decreased risk of diarrhea comparing continuous versus bolus administration;
**Prokinetics use**	Erythromycin should be intravenously administered (usually at dosages of 100–250 mg three times a day) as a first line prokinetic therapy; recommended to patients with gastric feeding intolerance; should be used for 24–48 h or maximum 4 days; if a large (>500 mL) GRV still persists, the use of post-pyloric feeding should be considered over withholding EN;
	Metoclopramide can be used alternatively, or in combination with erythromycin;
	Effectiveness of prokinetics is decreased after 3 days and should be discontinued;
**Energy expenditure (EE) estimation**	Several parameters must be considered in order to estimate caloric needs: the nutritional status prior to ICU admission (body weight and its alterations); the number of days of hospitalization before ICU admission and/or in the ICU; the endogenous nutrient production and autophagy; the energy balance during ICU stay; the time elapsed and energy balance during hospital stay; the occurrence of refeeding syndrome (or at least hypophosphatemia) at the time of feeding;
	Indirect calorimetry (IC) is recommended in critically ill mechanically ventilated patients;
	If IC is not available use V_O_2__ (oxygen consumption) from pulmonary arterial catheter or V_CO_2__ (carbon dioxide production) derived from the ventilator (REE = V_CO_2__ × 8.19);
	If IC and V_O_2__ or V_CO_2__ measurements are not available, use of simple weight-based equations (such as 20–25 kcal/kg/day);
**Isocaloric or hypocaloric nutrition therapy?**	Isocaloric nutrition if indirect calorimetry is used; after the early phase of acute illness can be progressively implemented; ^1)^ after day 3, caloric delivery can be increased up to 80–100% of measured EE;
	Hypocaloric nutrition (not exceeding 70% of EE), if predictive equations are used to estimate the energy need, should be administered in the early phase of acute illness especially for the first week of ICU stay;
**Energy/protein goal**	Should be achieved progressively and not before the first 48 h to avoid over-nutrition ^1)^;
	Full targeted medical nutrition therapy is considered to achieve more than 70% of the resting energy expenditure (REE), but not more than 100% ^1)^;
**Protein requirements**	1.3 g/kg protein equivalents per day (delivered progressively);
	1.2–1.5 g protein/kg/day in older people who are malnourished or at risk of malnutrition
**Carbohydrates requirements**	Should not exceed 5 mg/kg/min;
	Citrate (used in continuous veno-venous hemo-dia-filtration (CVVH)) increases carbohydrate load and should be included as a non-nutritional calorie intake;
**Fat requirements**	High-fat administration can lead to lipid overload and especially unsaturated fat to impaired lung function and immune suppression^1)^;
	The best ratio of fat can be established by monitoring of triglycerides and liver function tests;
	Propofol (lipid solution-1.1 kcal/mL) is associated with calorie overload;
**Glutamine (GLN)**	Additional enteral GLN should not be administered (exception: burn and trauma patients);
**EPA/DHA**	EN enriched with omega-3 FA within nutritional doses can be used;
	High doses omega-3 enriched enteral formulas should not be given on a routine basis and by bolus administration;
**Micronutrients**	Should be provided daily with PN;
	The repletion of micronutrients (i.e., Trace elements and vitamins) in conditions of chronic and acute deficiency is recommended;
**Antioxidants**	As high dose monotherapy should not be implemented without proven deficiency;
	Intense inflammation reduces the circulating levels (below reference ranges) of the antioxidant micronutrients (in particular copper, selenium, zinc, vitamins E and C);
**Vitamin D**	A high dose of vitamin D3 (500,000 IU) as a single dose within a week after admission can be supplemented if there are low plasma levels (25-hydroxy-vitamin D < 12.5 ng/mL, or 50 nmol/L);
**Supplemental PN**	In patients who do not tolerate full dose EN during the first week in the ICU, the safety and benefits of initiating PN should be weighed on a case-by-case basis ^1)^;
	PN should not be started until all strategies to maximize EN tolerance have been attempted ^1)^;
	When the level of energy needs provided by EN is below 60% 3 days after ICU admission, supplementary PN should be initiated to reach a maximum of 100% of the energy needs (measured by indirect calorimetry whenever possible) ^1)^;
**Obese and overweight patients**	An iso-caloric high-protein diet is recommended;
	Energy requirements are guided by indirect calorimetry (if not available use “adjusted body weight”*); protein requirements are guided by urinary nitrogen losses or lean body mass determination (if not available, 1.3 g of protein/kg “adjusted body weight”*/day);
	*“adjusted body weight” = (actual body weight-ideal body weight) × 0.33 + ideal body weight
	Ideal body weight for obese patients = 2.2 ×BMI + 3.5 × BMI × (height−1.5 m)
	Or use pragmatic approach: energy requirements = 20 ÷ 25% × (actual body weight-ideal body weight) + ideal body weight
	Early EN, gastrointestinal tolerance and progressive increase in nutrition recommended similarly as in all other ICU patients;
**Physical activity**	May improve the beneficial effects of nutritional therapy;
**Special conditions**	^1)^ EN should be delayed if shock is uncontrolled and hemodynamic and tissue perfusion goals are not reached, whereas low dose EN can be started as soon as shock is controlled with fluids and vasopressors/inotropes, while remaining vigilant for signs of bowel ischemia; in the case of uncontrolled life-threatening hypoxemia, hypercapnia or acidosis, whereas EN can be started in patients with stable hypoxemia, and compensated or permissive hypercapnia and acidosis; in patients suffering from active upper GI bleeding, whereas EN can be started when the bleeding has stopped and no signs of re-bleeding are observed; in patients with overt bowel ischemia; in patients with high-output intestinal fistula if reliable feeding access distal to the fistula is not achievable; in patients with abdominal compartment syndrome; if gastric aspirate volume is above 500 mL/6 h;
	^1)^ Low dose EN should be administered in patients: receiving therapeutic hypothermia and increasing the dose after rewarming, with intra-abdominal hypertension without abdominal compartment syndrome, whereas temporary reduction or discontinuation of EN should be considered when intra-abdominal pressure values further increase under EN; with acute liver failure when acute, immediately life-threatening metabolic derangements are controlled with or without liver support strategies, independent of grade of encephalopathy;
	^1)^ Early EN should be performed in patients: receiving ECMO (extracorporeal Membrane Oxygenation); with traumatic brain injury; with stroke (ischemic or hemorrhagic); with spinal cord injury; with severe acute pancreatitis; after GI surgery; after abdominal aortic surgery; with abdominal trauma when the continuity of the GI tract is confirmed/restored; receiving neuromuscular blocking agents; managed in prone position; with open abdomen; and regardless of the presence of bowel sounds unless bowel ischemia or obstruction is suspected in patients with diarrhea;
	^1)^ in non-intubated patients not reaching the energy target with an oral diet, oral nutritional supplements should be considered first and then EN;
	^1)^ in non-intubated patients with dysphagia, texture-adapted food can be considered; if swallowing is proven unsafe, EN should be administered;
	^1)^ in non-intubated patients with dysphagia and a very high aspiration risk, postpyloric EN or, if not possible, temporary PN during swallowing training with removed nasoenteral tube can be performed;
**Laboratory parameters monitoring**	Blood glucose after ICU admission or after nutrition therapy initiation and at least every 4 h, for the first 2 days in general;
	Insulin: when glucose levels exceed 10 mmol/L; insulin therapy initiation when blood glucose exceeds 150 or 180 mg/dL (10 mmol/L); blood glucose should target a concentration of 6–8 mmol/L;
	Electrolytes (potassium, magnesium, phosphate): at least once daily for the first week; refeeding hypophosphatemia: two to three times a day and supplemented if needed

^1)^ exact recommendations indicated in “ESPEN guideline on clinical nutrition in the intensive care unit” (2019).
